# Radiogenomics in Lymphoma and Multiple Myeloma: A Systematic Review of Current Evidence and Future Directions

**DOI:** 10.3390/jcm15114048

**Published:** 2026-05-23

**Authors:** Valentina Formica, Gayane Aghakhanyan, Valentina Baccolini, Francesca Pia Caputo, Salvatore Claudio Fanni, Roberto Francischello, Giuseppe Migliara, Duccio Volterrani, Riccardo Antonio Lencioni, Paolo Villari, Emanuele Neri, Dania Cioni

**Affiliations:** 1 Academic Radiology, Department of Surgical, Medical, Molecular Pathology and Emergency Medicine, University of Pisa, 56126 Pisa, Italy; valentina.formica@med.unipi.it (V.F.); riccardo.lencioni@unipi.it (R.A.L.); dania.cioni@unipi.it (D.C.); 2 Nuclear Medicine Unit, Department of Translational Research and of New Surgical and Medical Technology, University of Pisa, 56126 Pisa, Italy; duccio.volterrani@unipi.it; 3 Department of Public Health and Infectious Diseases, Sapienza University of Rome, 00185 Rome, Italy; valentina.baccolini@uniroma1.it (V.B.); paolo.villari@uniroma1.it (P.V.); 4 Academic Radiology, Department of Translational Research and of New Surgical and Medical Technology, University of Pisa, 56126 Pisa, Italy; francesca.caputo@med.unipi.it (F.P.C.); salvatoreclaudio.fanni@phd.unipi.it (S.C.F.); roberto.francischello@med.unipi.it (R.F.); emanuele.neri@unipi.it (E.N.); 5 Department of Life Sciences, Health and Health Professions, Link Campus University, 00165 Rome, Italy; g.migliara@unilink.it; 6 Italian Society of Medical and Interventional Radiology, SIRM Foundation, 20122 Milan, Italy

**Keywords:** radiogenomic, hematological malignancies, translational oncology, imaging-genomic integration, precision medicine

## Abstract

**Background/Objectives:** Radiogenomics integrates quantitative imaging features with genomic and molecular data to better characterize tumor biology and support precision oncology. While extensively investigated in solid tumors, its application to hematologic malignancies remains relatively unexplored despite the widespread use of advanced imaging in lymphoma and multiple myeloma. **Methods:** A systematic review was conducted following PRISMA 2020 guidelines. PubMed, Scopus, and Web of Science were searched up to December 2025 for studies investigating radiogenomic associations in hematologic malignancies. Study quality was assessed using PROBAST and METRICS. Two reviewers independently screened all records and performed data extraction through consensus. **Results:** Twelve studies were included, covering multiple myeloma and various lymphoma subtypes (aggressive B-cell lymphoma, classical Hodgkin lymphoma, and primary CNS lymphoma). Imaging modalities included PET/CT, MRI and CT. Across studies, radiomic and imaging-derived features were associated with cytogenetic abnormalities, gene expression profiles, and circulating tumor DNA metrics. In multiple myeloma, MRI and CT-based radiomics showed promising ability to predict high-risk cytogenetic abnormalities. In lymphoma, PET-derived volumetric and dissemination features correlated with molecular risk profiles and tumor microenvironment characteristics. Several studies demonstrated improved prognostic performance when imaging features were combined with genomic or clinical variables. **Conclusions:** Radiogenomic approaches in hematologic malignancies show promising potential for non-invasive risk stratification and improved prognostic assessment. However, current evidence remains limited by small cohorts, heterogeneous methodologies, and a lack of external validation. Prospective multicenter studies and standardized imaging–genomic pipelines will be essential to enable clinical translation.

## 1. Introduction

Multiple myeloma and lymphomas, including primary central nervous system lymphoma (PCNSL), constitute a biologically diverse subset of hematologic malignancies whose diagnosis, risk stratification, and treatment monitoring rely heavily on tissue or bone marrow biopsies, combined with cytogenetic and molecular analyses [1,2]. In recent years, next-generation sequencing (NGS) has further enriched diagnostic workups, enabling comprehensive profiling of recurrent mutations such as FLT3 and NPM1 in Acute Myeloid Leukemia (AML) or MYD88 and TP53 in lymphoma, which inform prognosis and therapy selection [3,4]. These methods remain the gold standard for defining the biological and genetic underpinnings of disease, providing essential insights for disease classification and therapeutic decision-making. However, these methods are invasive, time-consuming and may not fully capture the spatiotemporal heterogeneity of hematologic cancers [5,6,7].

Medical imaging, on the other hand, offers a complementary perspective by providing a non-invasive and repeatable assessment of disease burden and distribution across the whole body. Traditionally used for staging and response evaluation, imaging modalities such as computed tomography (CT), magnetic resonance imaging (MRI), and positron emission tomography (PET/CT) have become integral in the management of hematological malignancies [8,9]. In particular, [18F]FDG PET/CT is the cornerstone for staging and response-adapted therapy in lymphoma and is increasingly used in multiple myeloma, while CT and MRI continue to play essential roles in specific clinical contexts [10,11,12,13]. More recently, advances in computational image analysis have enabled the extraction of high-dimensional, quantitative features from medical images, a field known as radiomics. By turning medical images into computer-readable data, radiomics can reveal patterns that may relate to tumor biology and clinical outcomes [14,15]. When combined with machine learning (ML) and deep learning (DL), radiomics has demonstrated potential in refining risk stratification, treatment monitoring, and outcome prediction [16,17,18,19]. Early applications in hematological malignancies have shown that PET/CT radiomic features can predict outcomes in diffuse large B-cell lymphoma and multiple myeloma [20,21].

Building on these advances, the emerging field of radiogenomics seeks to integrate radiomic features with genomic, transcriptomic, and proteomic data, to identify associations between imaging phenotypes and tumor biology. Although often defined narrowly as the correlation of imaging features with genomic alterations, radiogenomics is increasingly considered in a broader sense to encompass transcriptomic and proteomic associations as well [22,23]. Radiogenomics aims to complement the traditional diagnosis approaches, offering a non-invasive and scalable means of linking molecular alterations with whole-body disease characteristics. In hematological malignancies, early studies have shown that radiomic features from [18F]FDG PET/CT can be linked to cytogenetic alterations in multiple myeloma [18] and integrated with molecular classifiers in lymphoma [20,24]. This integration could transform personalized oncology by bridging the strengths of both molecular and imaging domains, to improve diagnosis, classification, treatment selection, and prognostication [22].

While radiogenomics has been explored extensively in solid tumors, its application to hematological malignancies remains in its early stages. Most current studies are exploratory, retrospective, and limited in scale, but initial reports underscore feasibility and the promise of clinical translation [23]. Nevertheless, its potential is particularly compelling in this context, given the heterogeneity of hematologic cancers and the routine use of advanced imaging in their management. By capturing disease biology and distribution simultaneously, radiogenomic approaches may help overcome the limitations of either modality alone, offering a more comprehensive view of tumor biology and disease dynamics.

In this review, we summarize recent advances and emerging evidence on the application of radiogenomics in hematological cancers. Finally, we discuss the current challenges, opportunities, and future directions for radiogenomics in advancing precision medicine in hematologic malignancies.

## 2. Materials and Methods

A systematic review was conducted in accordance with the Preferred Reporting Items for Systematic Reviews and Meta-Analyses (PRISMA) (Supplementary File S1) guidelines and aims to synthesize current evidence on the integration of radiomics and genomics, collectively referred to as radiogenomics, in hematologic malignancies. The protocol for this systematic review was not registered.

### 2.1. Literature Search

A comprehensive literature search was performed across three major databases: PubMed, Scopus, and Web of Science. The search was conducted up to 10 December 2025, and included all studies published before that date. The following search string was used: (“radiogenomics” OR “imaging genomics” OR (“radiomics” AND “genetics”)) AND (“hematological malignancies” OR “lymphoma” OR “leukemia” OR “multiple myeloma” OR “Hodgkin” OR “non-Hodgkin” OR “hematologic cancers”). The search string included broad terms covering various disease entities (such as ‘leukemia’) to maximize literature retrieval and ensure maximum sensitivity across the spectrum of hematological malignancies, before applying the strict inclusion criteria. Only articles published in English were considered.

### 2.2. Eligibility Criteria

Studies were eligible for inclusion if they:

- Investigated hematologic malignancies (e.g., lymphoma, leukemia, multiple myeloma).
- Reported radiogenomic correlations, defined as the integration of imaging-derived features (quantitative radiomics, qualitative PET/MRI metrics) with genomic or molecular data.
- Presented original clinical data from cohort studies, prospective or retrospective designs.

The following were excluded:

- Studies involving only solid tumors or non-hematologic malignancies.
- Articles lacking either radiomic or genomic data.
- Case reports, reviews, or purely methodological articles.
- Conference abstracts, editorials, letters, and preclinical studies without clinical data.

### 2.3. Screening and Selection Process

All retrieved records were uploaded into Rayyan (https://www.rayyan.ai/ (accessed on 15 December 2025) an AI-assisted platform for systematic review management, where duplicates were automatically removed. Two independent reviewers screened titles and abstracts for relevance, and any disagreements were resolved by consensus discussions during joint meetings. Studies selected for full-text review were then assessed against the eligibility criteria.

### 2.4. Quality Assessmen Toolst

The quality of all included studies was assessed using the Prediction model Risk of Bias ASsessment Tool (PROBAST) [25]. This tool is specifically designed to evaluate risk of bias and applicability in studies that develop, validate, or update prediction models. To maintain consistency across the review, we applied PROBAST to all included studies, considering them as prediction model studies, whether diagnostic, prognostic or both, even in cases where a formally validated model was not developed. We evaluated them within this framework because they inherently assess the predictive capacity of imaging variables for genomic or clinical outcomes. Applying PROBAST allowed for a rigorous, standardized evaluation of biases related to participant selection, predictor measurement, and statistical handling across all studies without introducing the inconsistency of utilizing multiple distinct quality assessment tools. PROBAST comprises four domains:

1. Participants;
2. Predictors;
3. Outcome;
4. Analysis.

Each domain contains a structured set of signaling questions designed to guide the assessment process. These questions address specific aspects of study design, conduct and reporting that may introduce bias. Each signaling question was answered using one of the following response options: yes (Y), probably yes (PY), probably no (PN), no (N), or no information (NI). The responses to the signaling questions inform the judgment of the overall risk of bias within each domain, which is categorized as low, high, or unclear. An overall risk of bias across all domains is then assigned to the study. In accordance with PROBAST guidance, a study was judged to be at high risk of bias overall if any single domain was rated as high risk. In the context of PROBAST, applicability concerns were also assessed across three domains (participants, predictors, outcome) and classified as low, high, or unclear.

In addition to PROBAST, the methodological quality of radiomics studies was assessed using the METhodological RadiomICs Score (METRICS) tool, officially endorsed by the European Society of Medical Imaging Informatics (EuSoMII) [26]. METRICS is a structured, 30-item instrument with five conditional criteria designed to evaluate the robustness, transparency, and reproducibility of radiomics research, including both handcrafted and deep learning-based approaches. The METRICS assessment focused on methodological aspects unique to radiomics, such as segmentation methodology, feature extraction, dimensionality reduction, model validation, and open science practices, complementing the bias evaluation performed by PROBAST. Each study received a percentage score (0–100%) and a qualitative grade (Excellent ≥ 80%, Good 60–79%, Moderate 40–59%, Low < 40%), as recommended in METRICS-E3.

### 2.5. Data Extraction and Synthesis

Data were extracted into a standardized form, including study design, sample size, type of tumor, imaging modality, genomic data type, radiomic features analyzed, main radiogenomic associations, and clinical endpoints. Due to heterogeneity in imaging modalities, genomic data, and tumor types, a quantitative meta-analysis was not feasible; therefore, data were integrated and presented through a structured qualitative synthesis.

## 3. Results

### 3.1. Study Selection

**Figure 1:**
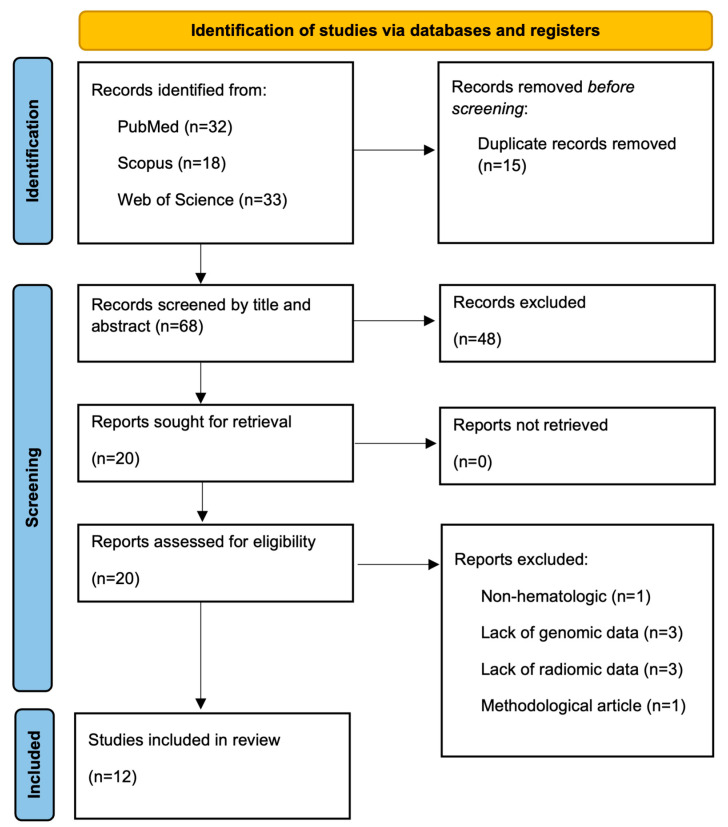
PRISMA flow diagram.

The initial database search yielded a total of 83 records. After removing 15 duplicates in Rayyan, 68 unique records remained for screening. Of these, 48 studies were excluded during the title and abstract screening as they did not meet the eligibility criteria. Notably, while our highly sensitive search string retrieved initial records related to leukemia, all of these were ultimately excluded at this stage or during the full-text review because they lacked the necessary integration of both radiomic and genomic data. The full text of the remaining 20 studies was assessed for eligibility, resulting in the exclusion of an additional 8 studies (1 non-hematologic, 3 lacking genomic data, 3 lacking radiomic data, 1 methodological article). Ultimately, 12 studies were included in the final synthesis. The study selection process is summarized in the PRISMA flow diagram (Figure 1).

### 3.2. Quality Assessment

As outlined in the Methods section, the risk of bias of all included studies (*n* = 12) was evaluated using PROBAST, considering them as prediction model studies with diagnostic, prognostic, or combined aims. Results of the domain-specific and overall assessments are summarized in Table 1. Overall, most studies showed low to moderate risk of bias across the four PROBAST domains (participants, predictors, outcome, and analysis). The analysis domain was the most frequent source of potential bias, often due to small sample sizes, limited handling of missing data or absence of external validation. In contrast, the participants and predictors domains were generally rated as low risk. Regarding applicability, all studies were judged to have low applicability concerns, except for Zhou et al. (2020) [27], which was rated as high concern due to issues in the participant selection and predictor definition. Taken together, the methodological quality of the included studies was considered adequate to support a comprehensive evaluation of the current literature, although caution is warranted when interpreting results from studies with high risk of bias in the analysis domain or with applicability concerns.

Complementary to PROBAST, the METRICS analysis provided a structured evaluation of the methodological rigor of the radiomics components across studies. As shown in Table 2 (studies listed in chronological order), the overall METRICS ranged from 35.1% to 98%, with most studies demonstrating good to excellent methodological quality. The highest scores were observed in studies employing highly standardized imaging pipelines, rigorous statistical validation and transparent feature extraction methods, e.g., Refs. [28,29,30].

Notably, S. Liu et al. (2025) [28] achieved the highest overall score of 98.0%. They utilized IBSI-compliant feature extraction software and applied robust feature reduction techniques such as maximum relevance minimum redundancy (mRMR) and LASSO. Crucially, they successfully validated their multi-sequence MRI nomogram in a strictly independent external cohort from a second institution. Similarly, Eertink et al. (2023) [29] excelled by utilizing IBSI-compliant feature extraction software. They also applied robust repeated cross-validation with internal feature selection to prevent optimism bias. Faghani et al. (2025) [30] was also categorized as “Excellent” due to its transparent handling of complex imaging data. Specifically, Faghani et al. rigorously validated their deep learning model via 5-fold cross-validation and occlusion saliency maps.

Studies categorized as “Good” [31,32,33,34] generally demonstrated solid radiomic workflows and robust classical multivariable modeling. For instance, Liu et al. (2021) [32] and Liu et al. (2021) [33] utilized appropriate feature reduction techniques (such as LASSO) and adhered to the recommended 1:10 dimensionality rule. However, these studies lost points primarily due to the lack of external validation datasets or, in some cases, the failure to compare their radiomic models against fully representative standard clinical risk scores.

Conversely, earlier works [27] or small pilot studies [35] showed limited reporting and lack of validation. These studies scoring in the “Low” category suffered from significant methodological limitations: Zhou et al. (2020) [27] relied entirely on univariate statistical comparisons without multivariable modeling. Meanwhile, Duffles et al. (2024) [35] exhibited severe dimensionality issues by entering multiple radiomic features into regression models for a cohort of only 18 patients, without applying internal validation techniques like bootstrapping or cross-validation.

### 3.3. Characteristics of Included Studies

The 12 included studies were published between 2009 and 2025 and were conducted in various clinical settings. As comprehensively summarized in Table 3, these articles employed a variety of imaging and genomic approaches to investigate radiogenomic associations in hematologic malignancies. While all studies combined molecular and imaging data to some extent, their methodologies differed substantially in disease focus, imaging modalities, genomic techniques, and analytical frameworks.

Key characteristics of the reviewed literature include:

- Tumor types: Four studies focused on lymphoma (including aggressive B-cell lymphoma, classic Hodgkin Lymphoma, and Primary CNS Lymphoma) [29,34,35,36] and eight on multiple myeloma [27,28,30,31,32,33,37,38].
- Sample sizes: Sample sizes ranged from 16 to 323 patients.
- Imaging modalities: Imaging modalities included PET/CT (*n* = 5), MRI (*n* = 6) and CT alone (*n* = 1).
- Genomic data: Genomic data encompassed gene expression profiling, cytogenetic abnormalities by FISH, mutational analysis via NGS panels, whole-genome sequencing, targeted DNA/RNA sequencing [28] and circulating tumor DNA (ctDNA) mutational profiles.

From a clinical perspective, the included cohorts predominantly consisted of newly diagnosed patients who were evaluated prior to the initiation of treatment. This trend highlights that current radiogenomic models are primarily being investigated as non-invasive tools for baseline risk stratification. A notable exception is the study by Zhou et al. (2020) [27] which specifically focused on a heavily pre-treated relapsed/refractory myeloma cohort. A summary of study characteristics is provided in Table 3.

#### 3.3.1. Imaging Modalities and Analysis

All studies integrated medical imaging into their analyses, but the choice of modality and the depth of image processing varied. Five studies used PET/CT as a core imaging technique [27,29,31,34,35], reflecting its central role in lymphoma and myeloma management. Zhou et al. (2020) [27] analyzed PET/CT more clinically, focusing on SUVmax and lesion counts/location. Conversely, Eertink et al. (2023) [29] and Durmo et al. (2022) [34] focused heavily on spatial dissemination metrics derived from PET/CT, such as the maximum distance between distant lesions (Dmax), alongside standard metabolic tumor volume (MTV) extractions. Bartel et al. (2009) [31] also employed PET/CT but complemented it with MRI and metastatic bone survey (MBS), providing a multimodal imaging assessment of multiple myeloma. Similarly, Zhou et al. (2020) [27] compared PET/CT findings with diffusion-weighted MRI in a subset of patients. Focusing strictly on MRI, Liu et al. (2021) [32], Liu et al. (2021) [33], Xiong et al. (2024) [38], and S. Liu et al. (2025) [28] extracted radiomic features from various spinal MRI sequences (T1WI, T2WI, FS-T2WI) to evaluate multiple myeloma spatial heterogeneity. J. Liu et al. (2025) [36] applied MRI radiomics to primary CNS lymphoma, and Wennmann et al. (2023) [37] utilized whole-body MRI. Faghani et al. (2025) [30] uniquely focused on whole-body low-dose CT (WBLDCT), leveraging deep learning (3D DenseNet-121) to classify cytogenetic risk groups directly from axial CT volumes.

#### 3.3.2. Genomic and Molecular Methods

The genomic techniques were also heterogeneous. Eertink et al. (2023) [29] utilized FISH to assess MYC, BCL2, and BCL6 rearrangements, while Durmo et al. (2022) [34] employed gene expression profiling to decode the tumor microenvironment. J. Liu et al. (2025) [36] focused specifically on BCL-6 rearrangements derived from pathological biopsy specimens. Similarly, Zhou et al. (2020) [27] analyzed cytogenetic risk markers by FISH and performed whole-genome sequencing in a subset of patients. Faghani et al. (2025) [30] Liu et al. (2021) [32], Liu et al. (2021) [33], Xiong et al. (2024) [38], S. Liu et al. (2025) [28], and Wennmann et al. (2023) [37] also focused on cytogenetic risk but used standard FISH on bone marrow aspirates. Bartel et al. (2009) [31] incorporated gene expression profiling and cytogenetics alongside imaging, enabling risk stratification into molecular subgroups. Uniquely, Duffles et al. (2024) [35] utilized liquid biopsies to extract circulating tumor DNA (ctDNA) and perform targeted NGS for mutational profiling and clonal evolution tracking.

#### 3.3.3. Statistical and Predictive Modeling

The statistical analysis frameworks varied considerably. Eertink et al. (2023) [29], Liu et al. (2021) [33], Xiong et al. (2024) [38], and J. Liu et al. (2025) [36] utilized robust cross-validated logistic regression models and nomograms. Durmo et al. (2022) [34] relied on multivariate Cox proportional-hazards models. Notably, S. Liu et al. (2025) [28] strengthened their modeling with an independent external validation cohort from a second medical center. Faghani et al. (2025) [30] and Wennmann et al. (2023) [37] applied cross-validated deep learning on CT and MRI images to classify cytogenetic risk. In contrast, Bartel et al. (2009) [31] primarily relied on conventional descriptive statistics, Kaplan–Meier survival curves and Cox models without advanced predictive modeling.

### 3.4. Radiogenomic Associations Reported

All included studies identified associations between radiomic features and genomic or molecular characteristics of hematologic malignancies, highlighting recurrent evidence of such correlations across different settings.

In aggressive B-cell lymphoma, Eertink et al. (2023) [29] demonstrated that a model combining [18F]FDG PET/CT radiomics (focusing on MTV and spatial dissemination) with MYC rearrangement status significantly outperformed standard clinical tools. This approach achieved a high positive predictive value (50.0%) for 2-year disease progression compared to the International Prognostic Index (29.6%). Further exploring DLBCL through liquid biopsies, Duffles et al. (2024) [35] found that baseline ctDNA levels significantly correlated with PET-derived TMTV, TLG, and radiomic textural features. Changes in these markers reflected clonal evolution and accurately predicted clinical remission.

In classical Hodgkin lymphoma (cHL), Durmo et al. (2022) [34] revealed that high lesion dissemination (Dmax > 20 cm) was an independent predictor of worse progression-free survival. This feature was also associated with an immune-depleted, macrophage-enriched tumor microenvironment, suggesting that a specific biological profile facilitates distant tumor dissemination.

In primary central nervous system lymphoma (PCNSL), J. Liu et al. (2025) [36] successfully developed a multi-sequence MRI-based radiomic nomogram to non-invasively predict BCL-6 rearrangements. They achieved an AUC of 0.871 in their validation set, demonstrating that spatial radiomic heterogeneity can reflect specific molecular subtypes in rare extranodal lymphomas.

In multiple myeloma, Bartel et al. (2009) [31] and Faghani et al. (2025) [30] reported complementary radiogenomic insights. Bartel et al. (2009) [31], in a large cohort of 239 patients, showed that [18F] FDG PET/CT features, such as the number of metabolically active focal lesions (FLs), presence of extramedullary disease (EMD), and high SUV, were independently associated with inferior survival. These features also correlated with adverse genomic profiles, including high-risk gene expression signatures. Notably, achieving PET negativity prior to transplant improved survival even among genomically high-risk patients, underscoring the prognostic synergy of imaging and molecular markers. Similarly, Faghani et al. (2025) [30] applied deep learning to whole-body low-dose CT (WBLDCT) in 151 newly diagnosed patients and demonstrated accurate prediction of cytogenetic risk groups. Their model revealed significant survival differences between predicted high- and standard-risk categories, providing further support for the utility of radiogenomic models in baseline risk stratification. Zhou et al. (2020) [27] further reinforced these findings in 24 patients with relapsed/refractory multiple myeloma. They showed that high-risk cytogenetic alterations (e.g., del(17p)/TP53 loss) were strongly associated with markedly elevated SUVmax (median 27 vs. 6) on PET/CT, and that SUVmax > 15 predicted significantly worse progression-free and overall survival. Together, these studies suggest that PET-derived metrics can serve as surrogates for underlying aggressive disease biology driven by specific genomic aberrations and underscore the prognostic value of PET beyond molecular profiling alone.

Expanding on multiple myeloma via MRI, several recent studies demonstrated the diagnostic strength of radiomics. Liu et al. (2021) [32] and Liu et al. (2021) [33] showed that spinal MRI radiomics could independently and reliably predict high-risk cytogenetic abnormalities (HRCAs), achieving AUCs above 0.82. Xiong et al. (2024) [38] further improved predictions by combining a seven-feature MRI radiomics signature with β2-microglobulin into a clinical-radiomic nomogram (AUC 0.82). S. Liu et al. (2025) [28] confirmed the translatability of this approach by validating a multi-sequence MRI-based nomogram in an independent external cohort (AUC 0.840). Conversely, Wennmann et al. (2023) [37] found that while deep learning and radiomics models accurately predicted plasma cell infiltration from pelvic MRI, they failed to reliably predict specific cytogenetic aberrations. This highlights the complexity and potential anatomical dependencies of radiogenomic correlations.

Overall, this evidence highlights that radiogenomics can capture both disease biology and spatial distribution in hematologic malignancies. Across diseases, MTV, SUVmax, lesion dissemination and the presence of EMD consistently emerged as imaging biomarkers of adverse prognosis, often reflecting high-risk genetic alterations or unfavorable gene expression profiles. Importantly, models that combine imaging features with clinical and genomic variables achieved superior predictive performance compared to unimodal approaches [28,29,30,31,32,33,38], underscoring the synergistic potential of integrative radiogenomics in advancing precision hematology.

## 4. Discussion

The evidence synthesized in this review suggests that radiogenomic approaches in multiple myeloma and various lymphoma subtypes are promising but still exploratory. By linking imaging phenotypes with genomic and molecular features, these approaches aim to extend beyond traditional imaging-based staging and may provide non-invasive markers of prognosis and treatment response. However, the evidence base remains limited, with heterogeneous study designs, relatively small patient cohorts, and variability in clinical endpoints. While some studies demonstrated strong methodological rigor, weaknesses identified by PROBAST and METRICS in earlier or smaller pilot studies limit the strength of broader conclusions. This underscores the need for standardized multicenter studies to establish robust and generalizable radiogenomic signatures.

The included studies highlighted the utility of advanced computational image analysis. Radiomics and deep-learning approaches applied to [18F]FDG PET/CT, MRI, or CT enable non-invasive, repeatable assessment of tumor burden and heterogeneity, making them attractive candidates to complement biopsy-based information. Methodologically, these imaging-based studies often applied direct predictive modeling approaches to successfully link macroscopic imaging phenotypes with underlying disease biology.

Comparisons across studies also revealed recurring patterns highly dependent on the underlying pathology. Magnetic Resonance Imaging (MRI) emerged as the most frequently utilized modality overall, dominating the evaluation of multiple myeloma and primary CNS lymphoma. Conversely, 18F-FDG PET/CT was the primary imaging tool for systemic lymphomas (aggressive B-cell and classic Hodgkin lymphoma), reflecting its central role in standard clinical staging. Cytogenetic profiling by FISH or NGS was a recurrent genomic reference standard, applied in myeloma [27,28,30,31,32,33,37,38] and CNS lymphoma [36].

The depth of analysis, however, varied substantially. While Eertink et al., (2023) [29] and Durmo et al., (2022) [34] implemented advanced radiomic feature extraction focusing on spatial dissemination, others relied primarily on standard imaging metrics such as SUVmax and lesion counts [27,31]. Similarly, only a subset of studies [29,30,37] incorporated machine learning or deep learning, whereas others used conventional statistics. Disease focus also shaped priorities: lymphoma studies [29,34,35,36] often emphasized spatial dissemination and tumor–microenvironment interactions, while myeloma studies [27,28,30,31,32,33,37,38] were more centered on bone disease burden and cytogenetic risk.

Taken together, these heterogeneous but complementary approaches illustrate both the conceptual promise and the current challenges of radiogenomics in hematologic malignancies. Advanced radiomic analysis [28,29,36,38], spatial dissemination and immune characterization [34], and AI-driven imaging models [30,37] each highlight different aspects of disease biology, but their combined evidence remains preliminary.

Importantly, the inclusion of studies with negative findings, such as Wennmann et al. (2023) [37], highlights the complexity of radiogenomic correlations. While several studies successfully predicted cytogenetic risk in multiple myeloma, Wennmann et al. demonstrated that MRI radiomics and deep learning failed to accurately predict cytogenetic aberrations in their specific pelvic cohort. This contrast underscores potential anatomical dependencies in imaging, the risk of publication bias, and the critical need for standardized protocols.

PROBAST frequently identified high risk of bias in the analysis domain, driven by small sample sizes, limited handling of missing data and lack of calibration or external validation. It is important to note that these high-risk ratings often reflect the stringent criteria of PROBAST when applied to studies not originally designed as formal prediction models. While these studies successfully identify significant radiogenomic associations, they frequently lack the statistical architecture required for predictive modeling. METRICS varied widely, reflecting inconsistent feature extraction, segmentation strategies, and reporting standards. This variability, together with the small number of available studies, limits generalizability and precludes any practice-changing conclusions.

From a clinical perspective, radiogenomics currently appears to be a hypothesis-generating tool that may refine risk stratification and treatment selection in selected scenarios supporting precision medicine in hematology. In newly diagnosed multiple myeloma, radiogenomic markers could complement ISS/R-ISS by capturing spatial heterogeneity beyond single-site bone marrow biopsies. In aggressive B-cell lymphoma, PET-derived volumetrics integrated with molecular risk factors (such as MYC rearrangement status) can drastically improve the identification of high-risk patients compared to standard clinical indices like the IPI. In PCNSL, radiogenomic associations remain very preliminary and require dedicated prospective studies. Prospective validation, harmonization of imaging protocols, and standardized reporting will be critical to translating these insights into practice.

Looking ahead, several directions appear critical for the maturation of radiogenomics in hematologic malignancies. Prospective multicenter studies with harmonized imaging acquisition standardized segmentation and feature-extraction pipelines will be essential to ensure reproducibility and comparability across cohorts. Importantly, external validation of predictive models, a major limitation of the included studies, was successfully addressed by S. Liu et al. (2025) [28] using an independent two-center cohort. The replication of such validation efforts and the development of open-access radiogenomic datasets will accelerate the transition from exploratory findings to robust biomarkers ready for prospective trials. Deeper integration of radiogenomics with other high-dimensional data, including liquid biopsy and circulating tumor DNA, could help capture tumor evolution more comprehensively and in real time.

While the majority of current radiogenomic literature in lymphomas relies on localized, static tissue biopsies, emerging proof-of-concept studies are pioneering a paradigm shift. For instance, Duffles et al. (2024) [35] successfully integrated PET/CT radiomics with ctDNA to dynamically track clonal evolution and whole-body tumor burden non-invasively. This dual non-invasive approach captures spatial and temporal heterogeneity much better than a single needle biopsy and represents a highly promising future direction for the field.

Harmonizing radiogenomic endpoints with established clinical frameworks, such as interim PET in lymphoma or risk stratification scores in multiple myeloma, will also facilitate clinical translation. As machine learning and deep learning methods continue to evolve, ensuring transparency, interpretability, and avoidance of bias will be vital to gain clinician trust and regulatory acceptance.

## 5. Conclusions

Radiogenomics in hematologic malignancies remains in an early stage of development but shows promising potential to complement existing diagnostic and prognostic tools. Across myeloma and various lymphomas, including PCNSL, imaging-derived features such as MTV, SUVmax, spatial dissemination and the presence of extramedullary disease have consistently correlated with high-risk genomic and molecular profiles. When combined with clinical and molecular data, radiogenomic models may achieve superior predictive performance compared with unimodal approaches. Future progress will depend on rigorous validation, standardization of methodologies, and deeper integration with molecular and immunological data. If these challenges are addressed, radiogenomics could progressively strengthen precision hematology by refining risk stratification, supporting treatment decisions, and potentially decreasing reliance on repeated invasive biopsies in selected settings.

## Figures and Tables

**Table 1:** Risk of bias and applicability assessment of included studies evaluated using the PROBAST tool. Legend: (+), low risk of bias or low concern regarding applicability; (-), high risk of bias or high concern regarding applicability; (?), unclear risk of bias or unclear concern regarding applicability.

Author, Year	Risk of Bias				Applicability			Overall	
	1. Participants	2. Predictors	3. Outcome	4. Analysis	1. Participants	2. Predictors	3. Outcome	Risk of Bias	Applicability
Zhou et al., 2020 [27]	-	-	+	?	-	+	+	-	-
S Liu et al., 2025 [28]	+	+	+	-	+	+	+	-	+
Eertink et al., 2023 [29]	+	+	+	+	+	+	+	+	+
Faghani et al., 2025 [30]	+	+	+	+	+	+	+	+	+
Bartel et al., 2009 [31]	+	+	+	-	+	+	+	-	+
Liu et al., 2021 [32]	+	+	+	+	+	+	+	+	+
Liu et al., 2021 [33]	+	+	+	+	+	+	+	+	+
Durmo et al., 2023 [34]	+	+	+	-	+	+	+	-	+
Duffles et al., 2024 [35]	+	+	+	-	+	+	+	-	+
J Liu et al., 2025 [36]	+	+	+	+	+	+	+	+	+
Wennmann et al., 2023 [37]	+	+	+	-	+	+	+	-	+
Xiong et al., 2024 [38]	+	+	+	-	+	+	+	-	+

**Table 2:** METRICS quality assessment of included studies.

Author, Year	Metrics (%)	Quality Category
Zhou et al., 2020 [27]	35.1	Low
S Liu et al., 2025 [28]	98	Excellent
Eertink et al., 2023 [29]	87.5	Excellent
Faghani et al., 2025 [30]	81.8	Excellent
Bartel et al., 2009 [31]	69.9	Good
Liu et al., 2021 [32]	75	Good
Liu et al., 2021 [33]	77.2	Good
Durmo et al., 2023 [34]	70.6	Good
Duffles et al., 2024 [35]	39.7	Low
J Liu et al., 2025 [36]	82.5	Excellent
Wennmann et al., 2023 [37]	76.7	Good
Xiong et al., 2024 [38]	84.8	Excellent

**Table 3:** Summary of articles.

Authors, Year	Patients	Tmor Type and Disease Status	Median Age (Range)	Imaging Modality & Radiomic	Genomic/Molecular Data	Main Findings
Zhou et al., 2020 [27]	24	R/R MM	Age: 62 (45–80)	18F-FDG PET/CT (SUVmax, lesion distribution, EMD)	FISH (HRCAs) and WGS (CD138+ cells)	HRCAs (del(17p)) linked to higher SUVmax and poor survival. WGS confirmed TP53 alterations linked to high metabolic activity.
S. Liu et al., 2025 [28]	195	MM (ND)	Age: Not specified	Spinal MRI (T1WI, T2WI, FS-T2WI); High-dimensional features were extracted and reduced to an optimal multi-sequence radiomic signature.	FISH (HRCAs)	Clinical-radiomic nomogram predicted HRCAs and was successfully validated in an independent external cohort (AUC 0.84).
Eertink et al., 2023 [29]	323	Aggressive BCL (ND)	Age: 63 (18–87)	18F-FDG PET/CT (17 features: SUV, MTV, TLG, 12 spatial dissemination features)	FISH (MYC, BCL2, BCL6 rearrangements)	Radiomics + MYC status outperformed IPI for 2-year progression (PPV 50%, AUC 0.77). High-risk profiles linked to larger MTV and dissemination.
Faghani et al., 2025 [30]	151	MM (ND)	Age: 64 (35–88)	WBLDCT (3D DenseNet-121 deep learning mode)	FISH (Trisomies, HRCAs)	DL accurately predicted cytogenetic risk, highest for t(4;14) (AUC 0.87). DL risk groups mirrored FISH-based survival outcomes. These findings support the model’s potential as a non-invasive tool for early risk stratification.
Bartel et al., 2009 [31]	239	MM (ND)	Age: 59 (33–75)	18F-FDG PET/CT, MRI, X-ray (Visual analysis: SUVmax, EMD, focal lesions)	Laboratory values, cytogenetics and GEP risk classification	PET/CT showed superior prognostic value. >3 focal lesions, EMD, and high SUVmax predict poor outcomes. PET negativity pre-transplant improves survival.
Liu et al., 2021 [32]	50	MM (ND)	Age: Not specified	Spinal MRI (T1WI, T2WI, FS-T2WI); 9 LASSO features (including first-order, GLSZM, NGTDM, and GLRLM features).	FISH (HRCAs)	MRI radiomics predicted HRCA (AUC 0.86). Combined with age/sex, AUC reached 0.87.
Liu et al., 2021 [33]	89	MM (ND)	Age: 61 (38–83)	3.0T MRI (T1WI, FS-T2WI); Top 3 LASSO features (including first-order, GLSZM, and GLRLM features).	FISH (HRCAs)	Logistic regression radiomics model best predicted HRCA (AUC 0.82). Adding clinical parameters did not improve performance.
Durmo et al., 2022 [34]	155	cHL (ND)	Age: 32 (16–79)	Baseline 18F-FDG PET/CT (MTV, TLG, SUVmax, Dmax)	Gene expression (NanoString) and CIBERSORTx	Dmax > 20 cm independently predicts shorter PFS. High Dmax associated with immunosuppressive, macrophage-enriched microenvironment.
Duffles et al., 2024 [35]	18	DLBCL (ND)	Age: 56 (22–85)	Baseline and post-tx 18F-FDG PET/CT (TMTV, TLG, SUVmax, textures)	ctDNA via targeted NGS (11 genes)	Baseline ctDNA correlated with TMTV/TLG. ctDNA clearance matched SUVmax drop and clinical remission. Driver mutations may persist.
J. Liu et al., 2025 [36]	102	PCNSL (ND)	Age: 66 (23–84)	Brain MRI (CE-T1WI, T2-FLAIR); high-dimensional radiomic features were extracted, and the most robust features were selected using LASSO to build a multi-sequence radiomics signature.	BCL-6 rearrangement (Biopsy)	CE-T1WI + T2-FLAIR radiomics predicted BCL-6. Clinical-radiomic nomogram (with age) achieved AUC 0.87.
Wennmann et al., 2023 [37]	285	SMM/MM (ND)	Age: 62 (29–87)	Whole-body pelvic MRI (T1, T2 STIR); 105 features by PyRadiomics + 3D DenseNet121 DL	FISH (HRCAs)	Models predicted plasma cell infiltration (AUC 0.86) but failed to predict cytogenetic aberrations (AUCs 0.49–0.61).
Xiong et al., 2024 [38]	151	MM (ND)	Age: 62 (36–84)	Lumbar MRI (FS-T2WI); 7 LASSO features (including first-order, GLCM, GLRLM, and GLSZM features).	FISH (HRCAs)	Radiomics predicted HRCAs (AUC 0.79). Combined nomogram (Rad-score + β2-microglobulin) achieved AUC 0.82.

## Data Availability

No new data were created or analyzed in this study. Data sharing is not applicable to this article.

## Supplementary Materials

The following supporting information can be downloaded at: https://www.mdpi.com/article/10.3390/jcm15114048/s1, File S1: PRISMA 2020 checklist [39].

## Abbreviations

The following abbreviations are used in this manuscript:

18F-FDG PET/CT	18F-fluorodeoxyglucose positron emission tomography/computed tomography
AI	artificial intelligence
AML	acute myeloid leukemia
AUC	area under the curve
BCL	B-cell lymphoma
CE-T1WI	contrast-enhanced T1-weighted imaging
cHL	classical Hodgkin lymphoma
CT	computed tomography
ctDNA	circulating tumor DNA
DL	deep learning
DLBCL	diffuse large B-cell lymphoma
Dmax	maximum distance/diameter
EMD	extramedullary disease
EuSoMII	European Society of Medical Imaging Informatics
FISH	fluorescence in situ hybridization
FLs	focal lesions
FS-T2WI	fat-suppressed T2-weighted imaging
GEP	gene expression profiling
GLCM	gray level co-occurrence matrix
GLRLM	gray level run length matrix
GLSZM	gray level size zone matrix
HRCAs	high-risk cytogenetic abnormalities
IPI	international prognostic index
LASSO	least absolute shrinkage and selection operator
MBS	metastatic bone survey
METRICS	METhodological RadiomICs Score
ML	machine learning
MM	multiple myeloma
MRI	magnetic resonance imaging
mRMR	maximum relevance minimum redundancy
MTV	metabolic tumor volume
MYC	myelocytomatosis oncogene
ND	newly diagnosed
NGS	next-generation sequencing
NGTDM	neighborhood gray-tone difference matrix
PCNSL	primary central nervous system lymphoma
PET/CT	positron emission tomography/computed tomography
PFS	progression-free survival
post-tx	post-treatment
PPV	positive predictive value
PRISMA	Preferred Reporting Items for Systematic Reviews and Meta-Analyses
PROBAST	Prediction model Risk of Bias ASsessment Tool
R/R	relapsed/refractory
Rad-score	radiomics score
SMM	smoldering multiple myeloma
STIR	short tau inversion recovery
SUVmax	maximum standardized uptake value
T1WI	T1-weighted imaging
T2-FLAIR	T2-weighted fluid-attenuated inversion recovery
T2WI	T2-weighted imaging
TLG	total lesion glycolysis
TMTV	total metabolic tumor volume
TP53	tumor protein 53
WBLDCT	whole-body low-dose computed tomography
WGS	whole-genome sequencing
